# Sleep after Heavy Alcohol Consumption and Physical Activity Levels during Alcohol Hangover

**DOI:** 10.3390/jcm8050752

**Published:** 2019-05-27

**Authors:** Lydia E. Devenney, Kieran B. Coyle, Thomas Roth, Joris C. Verster

**Affiliations:** 1 School of Psychology, Life and Health sciences, Ulster University, BT52 1SA Londonderry, Northern Ireland; Devenney-l2@ulster.ac.uk (L.E.D.); kb.coyle@ulster.ac.uk (K.B.C.); 2 Sleep Disorders and Research Center, Henry Ford Health System, Detroit, MI 48202, USA; troth@hfhs.org; 3 Division of Pharmacology, Utrecht University, 3584CG Utrecht, The Netherlands; 4 Centre for Human Psychopharmacology, Swinburne University, Vic 3122 Melbourne, Australia

**Keywords:** sleep, daytime activity, alcohol, hangover

## Abstract

Alcohol consumption can negatively affect sleep quality. The current study examined the impact of an evening of alcohol consumption on sleep, and next day activity levels and alcohol hangover. *n* = 25 healthy social drinkers participated in a naturalistic study, consisting of an alcohol and alcohol-free test day. On both days, a GENEactiv watch recorded sleep and wake, and corresponding activity levels. In addition, subjective assessments of sleep duration and quality were made, and hangover severity, and the amount of consumed alcoholic beverages were assessed. Alcohol consumption was also assessed in real-time during the drinking session, using smartphone technology. The results confirmed, by using both objective and subjective assessments, that consuming a large amount of alcohol has a negative impact on sleep, including a significant reduction in objective sleep efficiency and significantly lower self-reported sleep quality. Activity levels during the hangover day were significantly reduced compared to the alcohol-free control day. Of note, next-morning retrospective alcohol consumption assessments underestimated real-time beverage recordings. In conclusion, heavy alcohol consumption impairs sleep quality, which is associated with increased next day hangover severity and reduced activity levels. The outcome of this study underlines that, in addition to retrospectively reported data, real-time objective assessments are needed to fully understand the effects of heavy drinking.

## 1. Introduction

Alcohol hangover refers to the combination of mental and physical symptoms, experienced the day after a night of heavy drinking, starting when blood alcohol concentration (BAC) approaches zero [1]. Several factors may aggravate hangover severity and corresponding performance impairment, and one of them is the quality and duration of sleep after a heavy drinking session. Both hangover and sleep disturbances have shown to significantly impair potentially dangerous daily activities such as driving a car [2,3]. While people often report falling asleep immediately after alcohol consumption [4], the quality of sleep is often disturbed by the over production of glutamine [5]. Glutamine is a natural stimulant and alcohol produces both stimulant and sedative effects [6]. The stimulating effects of alcohol are thought to be associated with rising BACs (while drinking), whereas the sedative effects are associated with already high BAC levels [7]. The stimulating effects are linked to the activation of dopamine release in the brain’s ‘reward circuitry’ [6]. During alcohol consumption glutamine production is suppressed, and when alcohol leaves the body, the body then attempts to recover lost levels of glutamine. The increased glutamine levels after consumption has ceased and is referred to as glutamine rebound [8]. Roehrs et al. [9] found that when glutamine rebound occurs, increased waking and light sleeping was observed during the second half of the sleep period. On a normal night rapid eye movement (REM) and non-REM sleep periods alternate throughout the night with an average of six to seven cycles, however, after an evening of drinking this is reduced to two to three cycles [10]. It is therefore vital to further examine the relationship between alcohol consumption, sleep, and the alcohol hangover. Up to now, several studies have addressed this issue, and the collected evidence comes from either retrospective self-report or real-time assessments such as polysomnography.

### 1.1. Self-Report

Most evidence on the association between alcohol hangover and sleep comes from self-report, either gathered in clinical studies or via (retrospective) surveys. These revealed that drinking time often goes at the expense of total sleep time, and that alcohol has a detrimental effect on sleep quality. For example, in a controlled study, Finnigan et al. [11] observed that subjects fell asleep faster after alcohol consumption and reported reduced next-day alertness. McKinney and Coyle [12] examined alcohol hangover effects and sleep in 48 social drinkers. Applying a naturalistic study design, the researchers did not interfere with drinking behavior and no restrictions were placed on the subjects sleep behavior. Similar to Finnigan et al., McKinney and Coyle found that sleep was disrupted after alcohol consumption and next-day fatigue was significantly increased. After alcohol consumption, sleep was qualified as less satisfying, refreshing, and restful. Further, subjects went to bed significantly later when compared to the alcohol-free day, resulting in a significantly reduced total sleep time (TST). Moreover, with higher amounts of alcohol intake, sleep onset latency (time of falling asleep—time to bed; SOL) further reduced. Similar findings were reported by Hogewoning et al. [13] who’s naturalistic study revealed that drinking time goes at the expense of TST and that time-to-bed is significantly delayed by more than 1.5 h after alcohol consumption compared to an alcohol-free evening. 

Rohsenow et al. [14] examined powerplant performance in *n* = 61 merchant marine cadets the day following an evening of alcohol administration to achieve a BAC of 0.11%. Results were compared to an alcohol-free control test day. After an 8h period of supervised sleep, subjects reported significantly improved sleep quality in the alcohol condition. This unexpected finding may be explained by the fact that after alcohol consumption subjects reported significantly reduced sleep latency until sleep onset. Powerplant performance was not impaired in the hangover state.

Van Schrojenstein Lantman et al. [15] conducted a survey among 578 Dutch University students examining the impact of TST on the presence and severity of their past months latest alcohol hangover. Subjects who consumed more alcohol slept significantly longer. A positive correlation was found between TST and the duration of the alcohol hangover state. However, at the same time, prolonged TST was associated with significantly reduced overall hangover severity. Thus, reduced TST was associated with more severe hangover complaints. In a second survey by van Schrojenstein Lantman et al. [16], 335 adults reported that sleep quality was significantly worse after their latest alcohol consumption session that resulted in a hangover, and that daytime sleepiness was significantly increased compared to a regular alcohol-free day.

With regard to daytime activity, several studies revealed self-reports of increased apathy and hangover symptoms suggesting reduced activity during alcohol hangover [17,18].

### 1.2. Real-Time Assessments

In 2010, Rohsenow et al. applied polysomnography to examine sleep in relation to alcohol hangover in *n* = 95 social drinkers [19]. In a double-blind study, sleep was assessed after alcohol administration to achieve a BAC of 0.11% and an alcohol-free control day. Alcohol significantly decreased sleep efficiency and rapid eye movement sleep, and next-day self-reported sleepiness was significantly increased during hangover. Significantly worse hangovers were reported by subjects with reduced sleep efficiency and shorter TST. When hangover severity increased, less time was spent in rapid eye movement sleep. 

Earlier polysomnography studies with lower alcohol dosages revealed similar effects on sleep [9,20,21]. Alcohol significantly reduced sleep latency and the time spent in REM sleep. In the first half of the night, alcohol significantly increased the time spent in deep sleep (stage 3 and 4), while in the second half of the night, time spent in stage 1 sleep (drowsy light sleep) was significantly increased. The observations confirmed previous findings that after alcohol consumption people fall asleep quicker, spent less time in REM sleep in the first 4 h of sleeping [9]. The next 4 h, i.e., the second half of the night, sleep is more disturbed and fragmented, often characterized by multiple awakenings and increased time spent in Stage 1 sleep. Roehrs et al. [9] conducted a Multiple Sleep Latency Test (MSLT) the day following alcohol consumption (peak BAC 0.08%) or placebo. The assessments showed that throughout the post-alcohol day subjects were sleepier, as evidenced by the fact that they fell asleep significantly faster when compared to the alcohol-free day. 

More recently, Wilkinson et al. [22] applied actigraphy to a study with ten healthy subjects without sleep disturbances. Subjects continuously wore an actigraph, starting three nights before a day of alcohol consumption until 4 days thereafter. In the two days before the alcohol challenge, TST was on average 8.0 h and no naps were recorded. On the test day, at 9AM, alcohol was administered in a controlled laboratory setting to achieve a peak BAC of approximately 0.14%. Sleep behavior on the day and subsequent night were examined and next day (24 h after the start of alcohol consumption), subjects completed the Acute Hangover Scale [23]. Seven out of 10 subjects took an unscheduled afternoon nap, on average 8.7h after drinking, which lasted 0.6h. The authors further analyzed the data separately for those who napped (*n* = 7) and those who did not have a nap (*n* = 3) after alcohol consumption. The analysis revealed that the groups did not significantly differ on TST or hangover severity. Limitations of the study include its small sample size, and that alcohol was administered at 9AM in the morning. Therefore, it is unclear to what extend this study mimics real-life drinking and the ‘normal’ hangover experience. 

To our knowledge, physical activity levels during the hangover state have not been investigated previously. Additionally, real-time assessments of sleep and alcohol consumption are usually not conducted in hangover research. However, emerging research [24,25], provides a foundation in Ecological Momentary Assessment (EMA), which can be used to collect real time data. Here, EMAs were used to collect alcohol consumption measures every morning for 4–14 days [24] and one week [25]. Analysis of EMA data showed that subjects exceeded the threshold for binge drinking on drinking occasions [24]. The analysis also revealed more severe hangovers in adolescents (15–19 years) than adult heavy drinkers (21 or over) [24]. Moreover, severe hangover symptoms predicted less alcohol consumption on that particular day [24]. Using EMA messaging, Riordan et al. [25] showed that messages relating to short-term and long-term health and social consequences reduced alcohol consumption in female subjects but not males during orientation (freshers) week and semester 1. These studies [24,25] demonstrate the potential use of EMAs in both research and clinical interventions. 

Building on this, smartphone technology has been implemented to collect hourly intoxication ratings, and alcohol and water consumption. In addition the current study was conducted to examine sleep after an evening of heavy alcohol consumption and its relationship to next day hangover severity and physical activity. In order to closely mimic a real life drinking experience, the study had a naturalistic design in which the researchers monitored but did not intervene with alcohol consumption or other activities and behaviors, nor did the researchers control time-to-bed or wake up time. Thus, it provides a unique amalgamation of both the laboratory and naturalistic approach through the use of objective real time measures in a natural environment. 

Considering the literature presented above, it is predicted that alcohol consumption may not be accurately reported following a night of heavy drinking. It is also anticipated that sleep time will occur later in the evenings where alcohol is consumed. Finally, it is predicted that sleep efficiency and TST will be reduced during a hangover and participants will engage in less demanding physical activities during a hangover.

## 2. Methods

Twenty-eight healthy social drinkers (students of Ulster University) were recruited to participate in this naturalistic study. Participants were excluded for head injury, pharmaceutical treatment, pregnancy, and previous treatment for alcohol or drug abuse. Social drinking status, i.e., not being alcohol dependent was verified by self-report, and by completion of the Short Michigan Alcohol Screening Test (SMAST) [26]. Participants with a SMAST score greater than three were excluded from participation in the study. Ethical approval for this study was obtained from the ethics committee at Ulster University. All subjects provided written informed consent, and the study was conducted in accordance with the “Code of Ethics and Conduct” of the British Psychological Society (2009).

### 2.1. Design

The study comprised an evening of alcohol consumption and an alcohol-free (control) test day. Both experimental testing days occurred in free-living conditions whereby subjects did not have study or work commitments, or mandatory training to attend. Using a naturalistic study design, subjects consumed alcohol at a venue of their own choice, and the type and quantity of alcohol and activities during the evening were not controlled by the researchers in order to closely mimic real-life drinking occasions [6]. The investigators did not interfere with the participant’s activities and behavior. 

### 2.2. GENEactiv Accelerometer Assessments of Sleep and Activity

On each test day, participants were asked to wear, on their non-dominant hand, a GENEActiv accelerometer [27,28] to objectively assess activity levels and sleep. The GENEActiv accelerometer continuously records activity, environmental temperature and light exposure. The watch could not be operated by the participants, nor did they have access to the data collected. The device allows for raw data to be transferred wirelessly in real time and saved as an open source or csv. The data then can be analyzed in statistical packages such as SPSS and R [27,28]. Esliger et al. [29] has validated and calibrated the GENEActiv accelerometer using Metabolic Equivalent of Tasks (METs) and Signal Vector Magnitudes (SVM, magnitude of watch movement). METs represent the energy costs of physical activity [30]. One MET refers to an individual’s resting metabolic rate and can be calculated by dividing the volume of oxygen (VO_2_) used during the activity by 3.5 (1 MET = 3.5 mL O_2_/kg/min) [21,22]. The outcome intensity levels, categorized by Esliger et al. [29], and included in this study were: Sedentary (<1.5, METs), light (1.5–3.99 METs), moderate (4.00–6.99 METs), and vigorous (7+ METs) activity. The corresponding cut off points were set at 386 SVM (sedentary to light), 542 SVM (light to moderate), and 1811 SVM (moderate to vigorous). Outcome measures included the percentage of time spent in sedentary, light, moderate and vigorous activity from waking up to midnight, and total METs spent on the hangover and control day were calculated. Continuous measurements of activity level allowed calculation of time to bed, time of falling asleep, wake up time, TST, sleep efficiency (i.e., the ratio of total sleep time and the time spent in bed), and number and the median duration of nightly awakenings/activity. Using this data, sleep onset latency (time of falling asleep—time to bed) was computed.

### 2.3. Self-Reported Sleep (Next Morning)

Self-reported assessments of sleep comprised questions on time to bed, time of falling asleep, wake up time, sleep onset latency, and total sleep time. Using this information sleep efficiency was computed. To evaluate last night’s sleep, subjects rated several aspects of their sleep quality on a 7-point bi-polar scale ranging from extremely, quite, and slightly, around a midpoint of four (neither). Sleep quality was assessments by six bipolar ratings including good-bad, satisfying-not satisfying, restful-not restful, refreshing-not refreshing, and light-deep. This scale has previously been implemented successfully in hangover research [12] and was completed each test day in the morning.

### 2.4. Assessments of Alcohol Consumption (Real-Time and Retrospective)

On the day before the drinking session, a Droidsurvey/iSurvey app was installed on subjects’ smartphones, and they registered with the program (Harvest Your Data). Participants were identified through coded usernames and responses were recorded and synced to the researcher’s account online. When offline, the application stored data until the device went online. On the drinking occasion, real-time smartphone assessments of alcohol consumption were made. The app required touch screen responses to four short questions and took approximately 1 min to complete. Participants were asked to set reminders on their alarm to complete the app once hourly throughout their drinking episode. One of the questions assessed the amount of alcoholic beverages consumed during the past hour. 

The number of units of alcoholic beverages that was consumed last night was also assessed the following morning. To help recall and calculate the amount of beverages consumed pictures were shown with the drinks and corresponding standardized UK units that included wine, beer, alcopops, and shots of spirits (mixers). 

### 2.5. Assessments of Hangover Severity (Next Morning)

In the morning on each test day, the Acute Hangover Scale [23] was completed to measure the severity of 9 symptoms, including hangover, thirsty, tired, headache, dizziness/faintness, loss of appetite, stomach ache, nausea, and heart racing. Each item could be scored on a Likert scale ranging from 0 to 7, with the anchors none (0), mild (1), moderate (4), and incapacitating (7). The mean of the item scores represents overall hangover severity, with higher scores representing more severe hangovers.

### 2.6. Statistical Analysis

Statistical analysis was conducted with SPSS, version 24. Mean (SD) were computed for each variable. Results from the hangover day and control day were compared using paired sample *t*-tests, and in case the data was not normally distributed the nonparametric Mann-Whitney U test was applied. Differences were considered significant if *p* < 0.05. The relationship between objective and subjective sleep assessments, and other variables was investigated by computing Spearman’s Rank correlation coefficients, using difference scores (alcohol—control day). Correlations were considered significant if *p* < 0.05.

## 3. Results

Three participants did not attend the testing sessions, and as a result, 25 participants completed both testing sessions. On the experimental day, participants’ retrospectively reported a mean (SD) of 8.0 beverages (SD = 2.7), and their mean (SD) hangover severity was 2.2 (0.9). Descriptive statistics of the study sample are summarized in Table 1.

Results from the real time data collection of alcoholic drinks consumption revealed that a mean (SD) of 11.4 (3.8) beverages were consumed. However, participants reported a mean (SD) of 8.0 beverages (SD = 2.7) the following day. While the Pearson’s product-moment correlation revealed a significant association between alcohol consumption reported next day and in real time (*r* = 0.57, *p* < 0.01), a paired samples T-test revealed a significant difference in real time and next day reports of alcohol consumption (*t*(22) = −5.133, *p* = 0.0001).

### 3.1. GENEActiv Sleep Assessments

A summary of all GENEactiv sleep assessments in the hangover and control condition is given in Table 2.

The GENEactiv assessments further revealed that sleep efficiency was significantly worse after alcohol consumption (*p* = 0.04). Time to bed was significantly later on the alcohol and the control day. Wake up time did not significantly differ between the alcohol and control day. Interestingly, TST after alcohol consumption did not significantly differ from the alcohol-free control day. 

**Figure 1:**
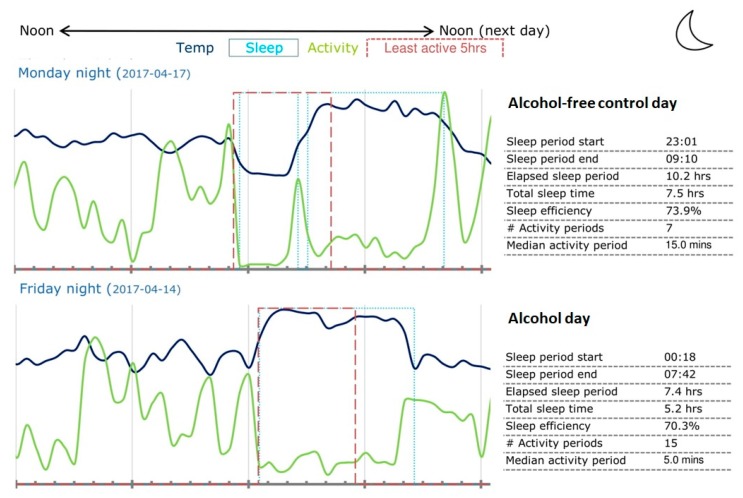
Visual output and summary data provided by the GENEActiv.

An example of the visual output and summary data is presented in Figure 1. It can be seen in Figure 1 that there is a delay in sleep time after alcohol consumption. Of note, drinking occurred on Friday evening.

The subject in this example went to bed 1 h and 17 min later after alcohol consumption when compared to the alcohol-free control night. In the control condition, 64% of participants went to bed at or after midnight and this was the case for 100% of participants on the alcohol test day. In line with this, TST was about 1.5 h shorter on the alcohol test day, confirming that usually drinking time goes at the expense of sleeping time. However, given the large variability in TST between subjects, the difference in TST between the alcohol and control test days did not reach statistical significance (*p* = 0.16).

### 3.2. Self-Reported Sleep

Self-reported sleep outcomes are summarized in Table 3. Ratings of sleep quality revealed that subjective quality of sleep was significantly worse after consuming alcohol than on the control day (*p* = 0.046), as well as significantly less restful (*p* = 0.001) and less refreshing (*p* = 0.01). Sleep was also rated more satisfying when participants did not consume alcohol, although the difference with the alcohol test day did not reach significance (*p* = 0.07).

### 3.3. Correspondence between Objective and Subjective Sleep Assessments

A Spearman’s R correlation analysis (using alcohol—control difference scores) comparing objective and subjective sleep measures revealed a significant negative relationship for TST (*r* = −0.41, *p* = 0.04). In addition, positive correlations were found between objective and subjective time of sleep onset (*r* = 0.71, *p* < 0.001) and wake-up time (*r* = 0.46, *p* = 0.02). There was no significant correlation between objective and subjective sleep efficiency, and paired *t*-tests revealed that this outcome significantly differed between the assessment methods (*p* = 0.03). Other sleep outcomes did not significantly differ between subjective and objective assessments. 

### 3.4. Correspondence between Sleep Assessments, Alcohol Consumption, and Hangover Severity

Spearman’s R correlation revealed that the total number of units of alcohol consumed and mean hangover severity did not significantly correlate with any of the sleep outcomes. 

### 3.5. Physical Activity

**Figure 2:**
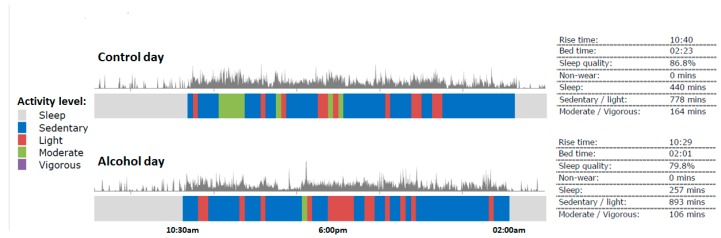
Physical activity levels on the alcohol and control day.

An example of physical activity assessment on the hangover day and the control day is given in Figure 2. It is evident from Figure 2 that activity levels were reduced on the hangover day. Most time was spent in the sedentary activity mode. In this example, moderate activity levels were seen on the control day, which were absent on the hangover day. It can be hypothesized that the absence of moderate activity levels are associated with the large reduction in total sleep time (i.e., more than 2 h in this subject) the night before the hangover day.

**Figure 3:**
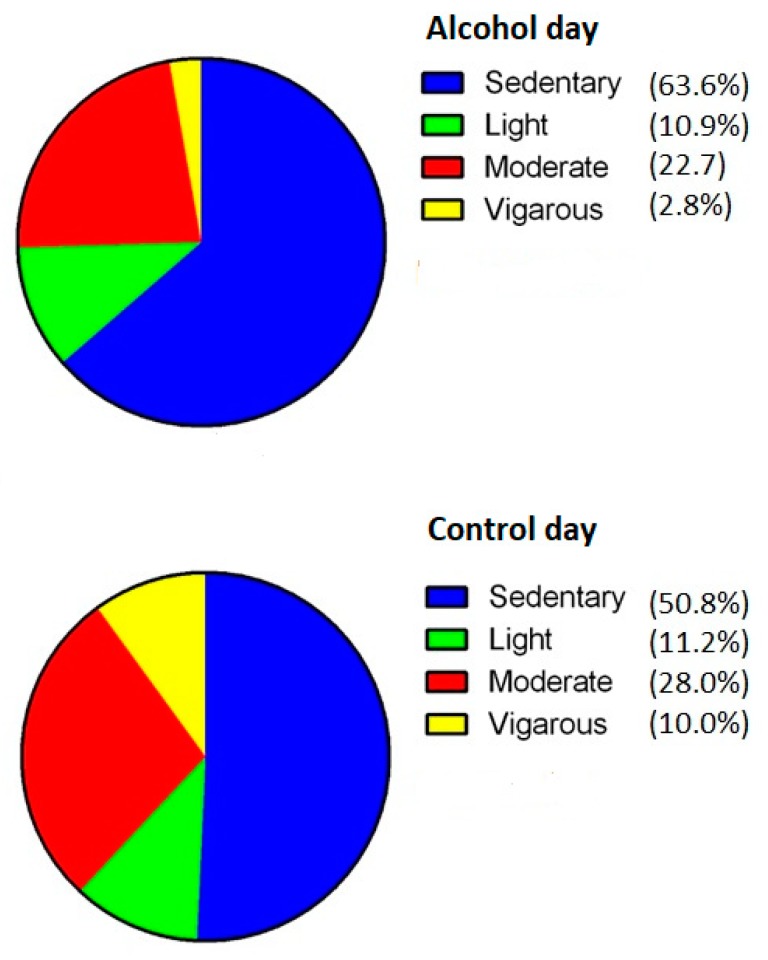
Percentage spent engaging in sedentary, light, moderate and vigorous activity on the alcohol day and control day.

The overall results on physical activity for the hangover and control day are summarized in Figure 3. First, the percentage of time spent on vigorous activity was significantly less (*p* = 0.03) on the hangover day (2.8%) compared to the control day (10.0%). Second, a significantly (*p* = 0.01) higher percentage of time was spent in a sedentary manner on the hangover day (63.6%) compared to the control day (50.8%). Third, no significant difference between the hangover and control day was observed for percentage of time spent on light activity (*p* = 0.86) and moderate activity (*p* = 0.09).

**Figure 4:**
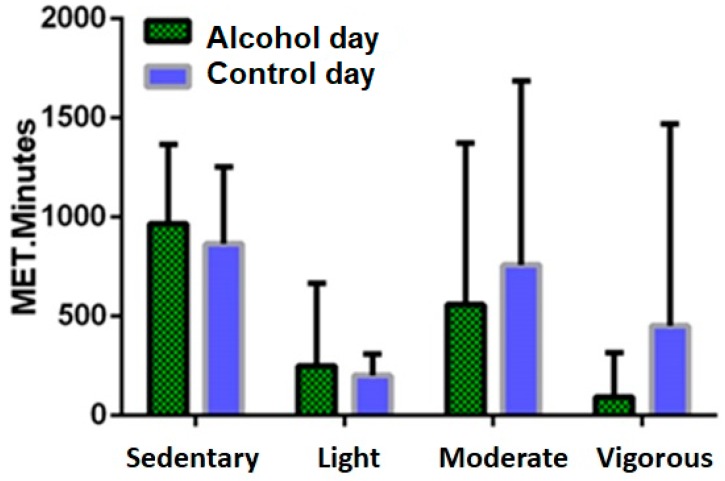
MET.minutes for different physical activity levels on the alcohol day and control day.

Figure 4 summarizes the amount of MET.minutes for different physical activity levels on the hangover day and control day. Total energy spent on the hangover day (1870 METs) was lower compared to the control day (2279 METs). However, the difference did not reach statistical significance (*p* = 0.37), presumably due to the large standard deviations observed on both the hangover and control day (SD = 1487 and SD = 1549, respectively). Wilcoxon Signed-Ranks analyses revealed no significant differences between hangover and control day for sedentary (*p* = 0.43), light (*p* = 0.62) or moderate (*p* = 0.06) MET.minutes. Thus, although a significantly less percentages of time was spent on sedentary activity on the hangover day compared to the control day, the level of energy spent at this level did not significantly differ between the test days. On the control day, the mean vigorous MET.minutes was significantly higher compared to the hangover day (*p* = 0.02).

### 3.6. Sleep and Drinking Variables Associated with Percentual Changes in Activity

A Spearman’s R correlation analysis was computed to compare the differences scores of percentages spent at the 4 activity levels (hangover-control day) with the total units of alcohol consumed, hangover severity, and sleep outcomes. Significant positive correlations were found between total units of alcohol consumed and differences in the percentage of light activity (*r*=0.45, *p* = 0.02). No significant association was found between percentual changes of activity levels and mean hangover severity. 

With regards to objective sleep measures, differences in percentages of sedentary activity positively correlated with wake-up time (*r* = 0.40, *p* = 0.049) as well as TST (*r* = 0.53, *p* = 0.01). Moreover MET.minutes of moderate activity negatively correlated with wake-up time (*r* = −0.44, *p* = 0.03). However, no other objective sleep measures correlated with MET.minute measurements. In terms of subjective sleep, the percentage of sedentary activity positively correlated with sleep onset latency (*r* = 0.52, *p* = 0.01) and moderate activity level MET.Minutes negatively correlated with sleep onset latency (*r* = −0.53, *p* = 0.01). Subjective measures of sleep quality revealed no significant differences relating to the percentage of activity levels or MET.minutes.

## 4. Discussion

Using both objective and subjective assessments, this study confirmed that consuming a large amount of alcohol has a negative impact on sleep, including a significantly reduced objective sleep efficiency and significantly lower subjective sleep quality. The study further revealed that next-day activity levels are significantly reduced during the alcohol hangover.

In relation to previous research, these results are in line with previous investigations of sleep and hangover [5,6]. Also in the current study, time to bed was significantly delayed by more than 1 h. However, in contrast to previous research [12,13,15], the difference in TST between the alcohol and control day in our study did not reach statistical significance, and in contrast to previous research [8,12] no significant association was found between objective TST and hangover severity. An explanation for the absence of significant effects may be the fact that there was great variability in TST between the subjects in this sample. Second, similar to previous findings by Rohsenow et al. [19], in the current study, sleep efficiency was significantly reduced after alcohol consumption. Whereas Rohsenow et al. [12] found a significant negative correlation between sleep efficiency and alcohol hangover, this correlation was not significant in the current study. In line with previous research [12,16], but not all [13], sleep quality was significantly poorer after alcohol consumption. Finally, several studies reported a reduced SOL after alcohol consumption [9,11,12,14]. While in the current study, subjective SOL was 20 min shorter after alcohol, the difference with the control day did not reach statistical significance. A correlation between alcohol consumption and hangover severity was not found. This observation supports recent findings [15] but contrasts with others [16]. It should be noted that the current study had less power than other studies that had a larger numbers of participants, so non-significant results can also be a result of low power for those particular analyses. 

### 4.1. Objective Versus Subjective Assessments

The objective and subjective sleep assessments were generally in agreement with each other. However, some discrepancies were also noted. Most notably, while after consuming alcohol objective TST was reduced by more than 1 h, the difference in subjective TST was much smaller (<30 min). A similar discrepancy was found for objective and subjective assessments of sleep efficiency. The reason for these differences in subjective and objective assessments are unclear, but it may be related that subjects are unaware of the number and duration of nightly awakenings. 

One could argue that these differences in outcomes underline the need for including real-time assessments to complement subjective sleep reports in clinical studies. Objective sleep may differ from perceived sleep and this discrepancy is not captured by relying solely on self-report. In this context, previous research in other areas has shown that subjects are sometimes unaware of performance or mood changes. For example, subjects were unaware of impairment in on-road driving tests after administering pharmacological treatment, while in contrast to this perception the objective assessments demonstrated that their actual driving performance showed clinically relevant impairment [31]. Thus, relying solely on patient perceptions of mood and impairment may therefore be dangerous in real life (e.g., they may decide to drive a car while they are not fit to drive) and bias clinical trial outcomes. For future research it is therefore recommended that, when possible, subjective ratings are complemented by objective assessments.

### 4.2. Recall Bias

When using a naturalistic study design, accurate capturing of alcohol consumption data is critical, as researchers are not present during the drinking session. It is therefore important to discuss the possible limitations of the methodologies used in this study.

While it should be taken into account that both being intoxicated and being hungover can result in inaccurate answering, this study revealed that retrospective reporting resulted in significant under-reporting of actual alcohol consumption. Retrospective self-report measures have long since been implemented in data collection. In the case of alcohol consumption this involves using retrospective memory to recall the type and number of alcoholic beverages consumed [32], as well as applying one’s ability to comprehend the question being asked, make decisions about the accuracy of the information recalled, and format an answer [33]. It is however unlikely that these factors may have played a role in the current student sample. Further, participants may be reluctant to disclose information about the number of drinks consumed the previous night. Additionally, this is unlikely to be an issue, as both research data and study participation was treated anonymously. The timing and place of data collection can however have had a significant impact on what is reported by study participants [34]. For example, diminished retrospective ability to recall the amounts of drinks consumed may be an issue, especially the morning after a night’s drinking when subjects suffer from an alcohol hangover. Research has shown that under these circumstances memory may be impaired [35,36]. Given this, accurately reporting alcohol consumption the day following a night’s drinking remains challenging, and is an issue inherent to conducting alcohol research. Furthermore, periods of memory loss while a person is intoxicated (e.g., blackouts or lapses of attention) can hinder both accurate retrospective recall [37] and real-time reporting. 

The use of smart phone and wearable technologies has garnered increasing attention in the fields of addiction research, as they offer the possibility of real time data collection at times people use alcohol and/or drugs in a natural setting and they also offer a more accurate assessment of alcohol consumption over time [38,39,40,41]. While real time data may be inaccurate because subjects are intoxicated, this data collection technique is not affected by recall bias. 

The integration of mobile phone technology to real time alcohol consumption data has been considered for some time [38]. While mobile phones were not as robust at the time, research concluded that it was a feasible alternative to paper and pencil self-monitoring [38]. Nonetheless, since then, few other studies have implemented smartphone technology to capture real-time alcohol consumption data and compare this to retrospective assessments [41,42,43]. Monk, Heim, and Price [41] applied smartphone technologies to investigate real-time alcohol consumption. Their application was designed to give hourly prompts to participants to select the context and number of drinks consumed. In line with our findings, their study confirmed that participants significantly under-report alcohol consumption when assessed by retrospective self-report measures. A difference of almost four drinks was reported on daily alcohol consumption (8.5 in real-time versus 4.2 retrospective). Taken together, these findings underline the importance of using real-time assessments when accurate real word evidence is needed.

### 4.3. Daytime Activity Levels

To our knowledge, this is the first study that directly compared objective assessments of levels of daytime activity during the hangover state and an alcohol-free control day. The assessments revealed that daytime activity levels were significantly reduced during alcohol hangover. These findings confirm previous self-reports of increased apathy and reduced alertness during the hangover state [16,17], which can be regarded as indications of reduced activity during alcohol hangover. While the GENEactiv watch objectively assessed the levels and duration of daytime activity, in the current study it was not assessed in what type of activity the subjects were actually engaged. Future research should address which specific activities are affected, delayed or fully skipped, and which other activities are conducted in the same manner as on an alcohol-free day. Also, motives for possible behavioral and activity level changes are largely unknown. Previous research has shown that potentially dangerous activities such as driving a car are significantly impaired during the hangover state [2]. It would be interesting to examine whether or not these types of behaviors are avoided or delayed during the hangover state, and how these behavioral changes and corresponding decision making are motivated by drinkers.

### 4.4. Limitations and Objectives for Future Research

The study has several limitations that should be addressed. First of all, there were great inter-individual differences in the data, which prevented several differences to reach statistical significance. While the study was adequately powered to demonstrate relevant differences between the hang over and control day, one should take into account that the hangover is a very personal experience that may vary from occasion to occasion. Hence, after consuming the same amount of alcohol, within a subject mood and cognitive effects and the impact on sleep may differ from drinking to drinking occasion. Therefore, future research should capture data from more than one drinking night to get a better overall view of the impact of alcohol consumption on sleep and subsequent hangover state. Second, not all assessments made by the GENEactiv watch were equally informative. For example, the standard output for nightly awakenings was the median duration of these awakenings. It would be more useful to have data on the frequency and duration of each individual nightly awakening. Unfortunately, with the current GENEactiv set-up, we could not recover this data. Third, while the assessments capture physical activity it would also be interested to collect objective data on mental activity during the alcohol hangover state. Electroencephalogram (EEG) and functional magnetic resonance imaging (fMRI) studies should be conducted to provide more insight on this topic. Up to now, only one small pilot study used fMRI to examine brain activity during alcohol hangover [44]. The authors reported that, in the hangover condition, subjects’ task performance was not significantly affected. However, maintenance of accurate performance level required compensatory increased activity of prefrontal and temporal brain structures. The authors concluded that correctly performing tasks in the hangover state requires significantly more mental effort compared to performing them on a normal non-drinking day. The interaction between physical and mental activity, and the mediating role of motivation and effort to accomplish tasks during hangover is a largely unexplored, an important topic for future research. 

## 5. Conclusions

The current study showed that sleep duration and quality is significantly negatively affected after alcohol consumption. Furthermore, during the hangover state, activity levels are significantly reduced. Our study advocates for implementing both objective and subjective assessments, and combine both real-time and retrospective measures to provide a more accurate view of a heavy drinking session, and its aftereffects.

## Figures and Tables

**Table 1:** Demographics and alcohol consumption characteristics.

	Mean (SD)
Demographics	
*n*	25
Male/Female	12/13
Age (years)	26.0 (7.1)
Age of first drink (years)	14.9 (1.7)
Usual Total sleep time (TST) (h:min)	6:33 (1:55)
Alcohol consumption on study night	
Reported units of alcohol consumed (real-time)	11.4 (3.8)
Reported units of alcohol consumed (retrospective)	8.0 (2.7)
Start time drinking (h:min)	20:48 (3:47)
Stop time drinking (h:min)	01:17 (1:12)
Duration of alcohol consumption (min)	269 (149.2)
Consumed more alcohol than planned (Yes/No)	10/15
Mean (SD) hangover severity	2.2 (0.9)

**Table 2:** GENEactiv sleep assessments.

Sleep Outcomes	Alcohol Day	Control Day	
	Mean (SD)	Mean (SD)	*p*-Value
Sleep start time	02:41 (1:17)	00.41 (1.16)	0.00 *
Wake-up time	9:46 (1:37)	8.56 (1:53)	0.07
Time in bed (h:min)	9:27 (2:46)	9:22 (2:14)	0.85
Total sleep time (TST) (h:min)	6:34 (3:45)	7:59 (4:42)	0.16
Sleep efficiency (%)	69.0 (16.7)	80.0 (15.2)	0.04 *
Number of nightly activity periods	8.4 (5.5)	8.0 (6.1)	0.81
Median duration nightly activity (min)	27.5 (59.9)	33.5 (79.0)	0.46

Significant differences (*p* < 0.05) between the alcohol and control test day are indicated by *.

**Table 3:** Self-reported sleep outcomes.

	Alcohol Day	Control Day	
	Mean (SD)	Mean (SD)	*p*-Value
Start time sleeping	02:28 (1:14)	00:23 (1:11)	0.00 *
Sleep onset latency (min)	29 (41)	49 (62)	0.22
Total sleep time (TST)	06:40 (1:53)	7:01 (1:50)	0.44
Wake-up time	09:00 (2:25)	8:25 (1:14)	0.00 *
Sleep efficiency (%)	94.5% (17.0%)	91.6% (8.6%)	0.55
Sleep quality ^1^			
Good-Bad	3.7 (1.6)	2.8 (1.3)	0.046 *
Satisfying-Not Satisfying	4.0 (1.6)	3.4 (1.3)	0.07
Refreshing-Not Refreshing	4.2 (1.4)	3.1 (1.2)	0.01 *
Restful-Not Restful	4.6 (1.3)	3.4 (1.4)	0.01 *
Light-Deep Sleep	5.0 (1.7)	5.0 (1.2)	0.92

Significant differences (*p* < 0.05) between the alcohol and control day are indicated by *. ^1^ Higher scores represent poorer sleep quality.
